# Supplementary material for: Parity-dependent State Engineering and Tomography in the ultrastrong coupling regime
Source: Sci Rep. 2015 Jul 8;5:11818. doi: 10.1038/srep11818 (PMC5155612; doi:10.1038/srep11818)
Supplement: Supplementary Information [file srep11818-s1.pdf]

Supplementary information

-

Parity-dependent State Engineering and Tomography in the ultrastrong coupling regime

S. Felicetti[1], T. Douce[2], G. Romero[1], P. Milman[2], and E. Solano[1,3]

[1]Department of Physical Chemistry, University of the Basque Country UPV/EHU, Apartado 644, E-48080 Bilbao, Spain
[2]Laboratoire Matériaux et Phénomènes Quantiques, Université Paris Diderot, CNRS UMR 7162, 75013, Paris, France
[3]IKERBASQUE, Basque Foundation for Science, Maria Diaz de Haro 3, 48013 Bilbao, Spain

1 Some properties of the Quantum Rabi model

We will not provide here a detailed description of the properties of the quantum Rabi model (QRM) eigenstates (instead we refer to [1]). The main feature we will focus on is the parity conservation of the QRM Hamiltonian. Let us define the parity operator $\Pi = -\sigma_z e^{i\pi a^\dagger a}$, which corresponds to the parity of the number of excitations in the composite system. Once the parity operator is defined, it is straightforward to show that it commutes with the QRM Hamiltonian. This ensures that any eigenstate of the quantum Rabi model is also an eigenstate of the parity operator.

Let us now consider the action of the creation operator on an arbitrary eigenstate $|\psi_n\rangle$. From its very definition, the creation operator creates one excitation inside the cavity, thus bringing $|\psi_n\rangle$ to a vector of opposite parity. In other words, $a^\dagger|\psi_n\rangle$ belongs to a subspace orthogonal to the one in which lies $|\psi_n\rangle$. Eventually we have shown the following relation:

$$\langle\psi_j|a^\dagger|\psi_n\rangle = 0 \tag{1}$$

for any j such that the parity of $|\psi_j\rangle$ is the same as $|\psi_n\rangle$, as shown in Fig.1. This demonstration naturally extends to the annihilation operator a. As a corollary, we have:

$$\langle\psi_n|a^{(\dagger)}|\psi_n\rangle = 0 \tag{2}$$

We show in the main text that apart from avoided crossings, the full model eigenstates are in product states made of eigenstates of the QRM and the ancilla being in the ground or excited state. Eq. (2) proves that the only contribution from the ancilla to the eigenenergies comes from the free Hamiltonian $(\omega_a/2)\sigma_z^a$. This explains the behavior of the eigenenergies as a function of ω_a: fully degenerate at $\omega_a = 0$, then increasing linearly.

2 Derivation of the effective interaction Hamiltonian

We consider the system as being composed of the the QRM interacting via the resonator field with the ancillary qubit. This means the total Hamiltonian reads:

$$\mathcal{H} = \sum_n \omega_n|\psi_n\rangle\langle\psi_n| + \frac{\omega_a}{2}\sigma_z^a + \mathcal{H}_I$$
$$\mathcal{H}_I = g_a\sigma_x^a(a + a^\dagger) \tag{3}$$

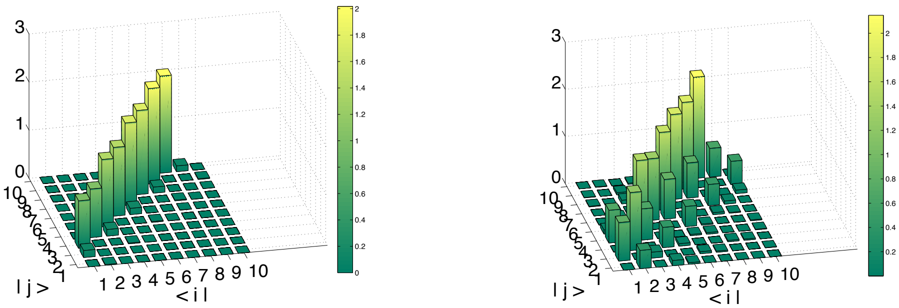

Figure 1: Absolute values of the elements of the transition matrix $k_{ij} = \langle\psi_i|a|\psi_j\rangle$. The left box corresponds to the strong coupling regime ($g/\omega_r = 0.03$), while the right box corresponds to the USC regime ($g/\omega_r = 0.6$), i.e. the quantum Rabi model. The diagonal term vanish, as shown in section 1. Notice that in the SC regime, where the Jaynes-Cummings model applies, the coefficients k_{ij} vanish for $\omega_i > \omega_j$.

where we denoted with $|\psi_n\rangle$ the eigenstates, of increasing energy ω_n, of the QRM. Using the completeness relation $\mathcal{I} = \sum_n |\psi_n\rangle\langle\psi_n|$, the interaction Hamiltonian becomes:

$$\mathcal{H}_I = g_a \sigma_x^a \sum_{i,j} k_{ij}|\psi_i\rangle\langle\psi_j| + k_{ji}^*|\psi_i\rangle\langle\psi_j| \tag{4}$$

where $k_{ij} = \langle\psi_i|a|\psi_j\rangle$. In the previous section we recalled that for any i we have $\langle\psi_i|a + a^\dagger|\psi_i\rangle = 0$. Thus we can order the double sum in equation Eq. (4) to get:

$$\mathcal{H}_I = g_a \sigma_x^a \sum_{i>j} (k_{ij} + k_{ji}^*)|\psi_i\rangle\langle\psi_j| + (k_{ji} + k_{ij}^*)|\psi_j\rangle\langle\psi_i| \tag{5}$$

Since the $|\psi_n\rangle$'s are labeled in increasing energy, we can interpret the two operators in the sum the following way: one raising the energy of the polariton, $|\psi_i\rangle\langle\psi_j|$, the other one lowering the energy $|\psi_j\rangle\langle\psi_i|$. Now we will assume that the spectrum of the QRM is non-linear enough so that we are able to isolate one particular transition frequency $\omega_{ij} = \omega_i - \omega_j > 0$. This anharmonic assumption is valid in the regime of $g/\omega_r \lesssim 2$, which is the one we consider here. Thus we can perform a new Rotating Wave Approximation (RWA) when bringing the frequency of the ancilla close to resonance with ω_{ij}. More precisely, we move $\mathcal{H}_I$ to the interaction picture:

$$\tilde{\mathcal{H}}_I(t) = g_a(\sigma_+^a e^{i\omega_a t} + \sigma_-^a e^{-i\omega_a t})((k_{ij} + k_{ji}^*)|\psi_i\rangle\langle\psi_j|e^{i\omega_{ij}t} + (k_{ji} + k_{ij}^*)|\psi_j\rangle\langle\psi_i|e^{-i\omega_{ij}t}) \tag{6}$$

In this expression we identify two oscillating frequencies: $\omega_a + \omega_{ij}$ and $\omega_a - \omega_{ij} \equiv \delta$. In this context we will perform the standard RWA, neglecting the quickly oscillating terms. The interaction picture Hamiltonian reads:

$$\tilde{\mathcal{H}}_I(t) = g_a((k_{ij} + k_{ji}^*)\sigma_-^a|\psi_i\rangle\langle\psi_j|e^{-i\delta t} + (k_{ji} + k_{ij}^*)\sigma_+^a|\psi_j\rangle\langle\psi_i|e^{i\delta t}) \tag{7}$$

thus yielding a Jaynes-Cummings-like interaction Hamiltonian.

3 Estimation of the time required to perform the spectroscopy protocol

Our protocol allows for analyzing the spectrum of the polariton, based on measurements performed on the ancillary qubit. This means the relevant parameters for this protocol are well-known and the manipulations are now standard [2]. In this section we will provide a rough estimation of the time required to detect the peaks in Fig. 4 of the main text.

The right order of magnitude for the experimental ω_2 spacing is given by the full width at half maximum (FWHM) of those peaks. In our case, the FWHM is of the order of $0.1\omega_r$. Besides, we span with ω_r an interval of approximate

length $2\omega_r$. Considering 5 points per peak, we obtain an upper bound on the number of points we want to measure of 100. This value could be further reduced performing a more clever analysis of the spectrum.

Every point actually corresponds to computing the visibility of Rabi oscillations at a given ancilla frequency. This can be done by measuring the ancilla until half a period, in other words by monitoring the ancilla for a time $T_{\rm half} \approx 50/\omega_r$ (see Fig. 4c in the main text). This monitoring requires to measure σ_z^a roughly 50 times, every measurement being of a duration at most $T_{\rm half}$. For a standard cavity in circuit QED, we have $\omega_r \approx 2\pi \times 5$ GHz, which gives approximately 100 ns to recover the visibility at a given frequency ω_2.

Going from one point to another means tuning the ancillary qubit frequency. This can be done in a few nanoseconds [2], hence it is negligible compared to the computation of a single point. In the end, summing 100 ns for 100 values of ω_2, the whole spectroscopy duration is of the order of 10 microseconds.

4 Multi-step process for state engineering and tomography

In the main text we show how selective state-transfer processes allow to prepare and manipulate the USC system in a given state, and to perform quantum state tomography. In the theoretical framework that we introduced, selective state transfer which involves USC system states of different parity are straightforward, as the ancilla spin-flip grants parity conservation. The goal of this section is to show how one may address transitions that should be forbidden because of parity conservation, by means of a feasible two-step protocol. Let $|\psi_a\rangle$ and $|\psi_b\rangle$ two eigenstates of the QRM of same parity. We want to distinguish the pure states made of an arbitrary superposition of $|\psi_a\rangle$ and $|\psi_b\rangle$ from the statistical mixture with same weights. To this end, we will make use of an auxiliary eigenstate of the QRM with opposite parity $|\phi\rangle$. In the following we will consider two different cases: first when the energy of $|\phi\rangle$ lies in between the energies of $|\psi_a\rangle$ and $|\psi_b\rangle$, then when it does not.

4.1 Forbidden transition with an auxiliary one in between

We suppose here the following ordering of the Rabi eigenstates energies. Namely: $E_{|\psi_a\rangle} < E_{|\phi\rangle} < E_{|\psi_b\rangle}$. We will consider that only those three levels are populated, assuming that we are able to initialize the ancilla in the $|+\rangle \equiv (|e\rangle + |g\rangle)/\sqrt{2}$ state. The initial state reads:

$$|\phi_i\rangle = (\alpha|\psi_a\rangle + \beta|\psi_b\rangle + \gamma|\phi\rangle)|+\rangle \tag{8}$$

First we perform half a Rabi oscillation between $|\psi_a\rangle$ and $|\phi\rangle$. This transforms the global state as follows:

$$|\phi_1\rangle = \alpha\frac{|\psi_a\rangle|g\rangle + |\phi\rangle|g\rangle}{\sqrt{2}} + \beta|\psi_b\rangle|+\rangle + \gamma\frac{|\psi_a\rangle|e\rangle + |\phi\rangle|e\rangle}{\sqrt{2}} \tag{9}$$

Then we repeat the protocol for the transition between $|\phi\rangle$ and $|\psi_b\rangle$. Thus we have:

$$|\phi_2\rangle = \alpha\frac{|\psi_a\rangle|g\rangle + |\phi\rangle|g\rangle}{\sqrt{2}} + \beta\frac{|\phi\rangle|e\rangle + |\psi_b\rangle|e\rangle}{\sqrt{2}} + \gamma\frac{|\psi_a\rangle|e\rangle + |\psi_b\rangle|g\rangle}{\sqrt{2}} \tag{10}$$

which we can write in a more convenient way:

$$|\phi_2\rangle = \frac{1}{\sqrt{2}}(|\phi_a\rangle|g\rangle + |\phi_b\rangle|e\rangle) \tag{11}$$

where $|\phi_a\rangle$ and $|\phi_b\rangle$ are two non-orthogonal states. Eventually, the reduced density matrix of the ancillary qubit reads:

$$\rho_a = \begin{pmatrix} |\alpha|^2 + \frac{1}{2}|\gamma|^2 & \frac{1}{2}(\alpha\beta^* + \alpha\gamma^* + \gamma\beta^*) \\ \frac{1}{2}(\alpha^*\beta + \alpha^*\gamma + \gamma^*\beta) & |\beta|^2 + \frac{1}{2}|\gamma|^2 \end{pmatrix} \tag{12}$$

Finally, assuming that we can infer the coherences between $|\psi_a\rangle$ and $|\phi\rangle$ and between $|\psi_b\rangle$ and $|\phi\rangle$ separately, this protocol allows for measuring the coherence terms relative to a forbidden transition – which correspond to the product $\alpha\beta^*$.

4.2 Forbidden transition between two consecutive eigenstates

In the case where the forbidden transition involves two consecutive eigenstates the result is a bit different. The energies correspond to $E_{|\psi_a\rangle} < E_{|\psi_b\rangle} < E_{|\phi\rangle}$. The initial state is again

$$|\phi_i\rangle = (\alpha|\psi_a\rangle + \beta|\psi_b\rangle + \gamma|\phi\rangle)|+\rangle \tag{13}$$

The first excitation transfer, associated with the transition between $|\psi_a\rangle$ and $|\phi\rangle$ yields:

$$|\varphi_1\rangle = \alpha\frac{|\psi_a\rangle|g\rangle + |\phi\rangle|g\rangle}{\sqrt{2}} + \beta|\psi_b\rangle|+\rangle + \gamma\frac{|\psi_a\rangle|e\rangle + |\phi\rangle|e\rangle}{\sqrt{2}} \tag{14}$$

Then when it comes to the transition between $|\psi_b\rangle$ and $|\phi\rangle$ the global state becomes:

$$|\varphi_2\rangle = \alpha\frac{|\psi_a\rangle|g\rangle + |\psi_b\rangle|e\rangle}{\sqrt{2}} + \beta\frac{|\phi\rangle|e\rangle + |\psi_b\rangle|e\rangle}{\sqrt{2}} + \gamma\frac{|\psi_a\rangle|e\rangle + |\phi\rangle|e\rangle}{\sqrt{2}} \tag{15}$$

which we can once again write, defining two non-orthogonal states $|\varphi_a\rangle$ and $|\varphi_a\rangle$ different than before:

$$|\varphi_2\rangle = \frac{1}{\sqrt{2}}(|\varphi_a\rangle|g\rangle + |\varphi_b\rangle|e\rangle) \tag{16}$$

and for the reduced density matrix of the ancilla:

$$\rho_a = \begin{pmatrix} |\beta|^2 + \frac{1}{2}|\alpha|^2 & \frac{1}{2}(\alpha\gamma^* + \beta\alpha^* + \beta\gamma^*) \\ \frac{1}{2}(\alpha^*\gamma + \beta^*\alpha + \beta^*\gamma) & |\gamma|^2 + \frac{1}{2}|\alpha|^2 \end{pmatrix} \tag{17}$$

Once again, one may access the coherence terms relative to the forbidden transition.

References

[1] D. Braak, Phys. Rev. Lett. **107**, 100401 (2011).

[2] S. J. Srinivasan, A. J. Hoffman, J. M. Gambetta, and A. A. Houck, Phys. Rev. Lett. **106**, 083601 (2011).
